# Methodological Pitfalls of Monitoring: Water Conditions Affect the Efficiency of Bottle Traps and Capture Success

**DOI:** 10.3390/biology14101416

**Published:** 2025-10-15

**Authors:** Teodor J. Purger, Boldizsár Szűcs, József Dezső, László Wágner, Dragica Purger, Jenő J. Purger

**Affiliations:** 1 Department of Ecology, Institute of Biology, Faculty of Sciences, University of Pécs, Ifjúság útja 6, H-7624 Pécs, Hungary; teodor.purger@gmail.com (T.J.P.); szubola95@gmail.com (B.S.); 2 Department of Physical and Environmental Geography, Institute of Geography and Earth Sciences, Faculty of Sciences, University of Pécs, Ifjúság útja 6, H-7624 Pécs, Hungary; dejozsi@gamma.ttk.pte.hu; 3 Duna-Drava National Park Directorate, Tettye tér 9, H-7625 Pécs, Hungary; waglaci@gmail.com; 4 Institute of Pharmacognosy, Faculty of Pharmacy, University of Pécs, Rókus utca 2, H-7624 Pécs, Hungary; dragica@ttk.pte.hu; 5 BioRes Limited Partnership, Barackvirág utca 27, H-7624 Pécs, Hungary

**Keywords:** hungary, *Lyssotriton vulgaris*, monitoring, sex ratio, *Triturus dobrogicus*, *Umbra krameri*, wetland, water supply

## Abstract

**Simple Summary:**

The Szaporca Old-Drava oxbow, one of Hungary’s earliest wetlands designated under Ramsar Convention, has faced significant threats due to drying. This necessitated the implementation of water replenishment measures. Our study aimed to assess the assemblage of aquatic vertebrates detectable by using bottle traps and to examine how water replenishment influences the traps’ efficiency and capture success. We found that the relative frequency of smooth newts caught with bottle traps was 10%, while that of other amphibian and fish species did not even reach 1%. Following the increase in water volume in the oxbow, both the efficiency of bottle traps (measured as the number of traps that captured smooth newts) and their capture success (number of caught newt individuals) declined significantly. The proportion of male smooth newts caught in traps increased significantly relative to females. Water replenishment also altered flow conditions and increased the extent of inundated areas, which in turn changed the substrate beneath the bottle traps. These environmental changes likely contributed to variations in trap performance. Our finding suggests that both water volume and associated habitat modifications can significantly influence the outcome of monitoring efforts using passive trap methods.

**Abstract:**

Wetland diversity and associated wildlife is declining globally. The Szaporca Old-Drava oxbow, one of Hungary’s first wetlands designated under the Ramsar Convention, has been threatened by desiccation, prompting the implementation of water replenishment interventions. This study aimed to determine which aquatic vertebrate species can be detected using bottle traps in the oxbow and whether the traps’ efficiency and capture success change following hydrological restoration. Our results showed that the relative frequency of smooth newts caught with bottle traps was 10%, while that of other amphibians (Danube crested newt, common spadefoot toad, edible frog) and fish (European weather loach, European mudminnow, Danube whitefin gudgeon) species did not even reach 1%. Based solely on the relative frequency data of the smooth newt, we found that both the capture efficiency (10.4% vs. 3%) and capture success (17% vs. 7.4%) of bottle traps significantly declined following an increase in water volume in the oxbow. Sex ratio patterns also shifted markedly: in the year of water scarcity, the male-to-female ratio of smooth newt was 2.7:1, whereas during water-abundant conditions it increased to 7:1. Water replenishment raised the water level by nearly one meter, resulting in the partial inundation of terrestrial vegetation and consequent habitat alterations. While water supplementation clearly supports the persistence of rare, protected, strictly protected and endemic aquatic species, our findings highlight the importance of considering hydrological conditions when interpreting the results of long-term monitoring in wetland ecosystems.

## 1. Introduction

Freshwater habitats are increasingly threatened by anthropogenic activities and climate change, often leading to their disappearance along with the extinction of species living there [1,2]. Their biodiversity is in crisis on a global scale [3] with a quarter of aquatic fauna currently threatened with extinction [4]. Amphibians, which rely on wetlands for reproduction, are particularly vulnerable to drought and environmental changes [5,6,7]. Their population decline results from a combination of different abiotic and biotic factors with effects that are context dependent. Consequently, even different populations of the same species may respond differently to a given environmental disturbance, so the causes of population decline can vary over space and time [8,9,10]. Because of these sensitivities, amphibian species are considered to be indicators of general environmental health. Understanding their population changes can serve as a model for interpreting monitoring data for other threatened species [11].

The drastic reduction in freshwater habitats in Europe is particularly pronounced in the Carpathian Basin [12,13,14]. Over the last centuries large rivers were regulated, and floodplains were drained to expand agricultural areas [15,16]. Recently, Central and Eastern European countries have become extremely vulnerable to climate change [17]. The water demand of agriculture has risen, pollution and disease outbreaks have become more frequent, and an increasing number of invasive species are spreading [18]. These trends negatively affect wetlands in Hungary highlighting the urgent need for habitat restoration [19,20]. Large-scale floodplain rehabilitation projects have been carried out in the floodplain of the Drava River, aiming to restore the water supply to oxbow lakes and side arms [21,22,23]. One of Hungary’s first wetlands designated under the Ramsar Convention is the Szaporca Old-Drava oxbow, which formed after a section of the Drava River, approximately ten kilometers long, had been cut off during river regulation [22,24]. This oxbow gradually lost its natural water supply, and water shortage became a serious problem [25] that has been exacerbated in recent decades by advanced vegetation succession and climate change. Such habitats can be maintained by periodic dredging and artificial water replenishment [21]. The Szaporca Old-Drava oxbow has been threatened by desiccation, leading to the implementation of water replenishment interventions. This oxbow was previously known primarily for its ornithological significance [26,27], but only sporadic faunal data existed on the occurrence of other vertebrates, such as fish or amphibians [25,28]. Notably, the rare endemic European mudminnow [28] and Danube crested newt [25], which were detected in this oxbow, have hardly any proven occurrence data from Southern Transdanubia region [29,30,31]. Since the lack of knowledge of their populations it is essential to monitor how they respond to water replenishment efforts. Bottle traps are commonly used to detect and monitor relative abundance of newts [32,33] and are also suitable for capturing other amphibians and fish [34]. The sex ratio of captured newts is characterized by the shift on the benefit of males or females [35,36,37,38]. This phenomenon, that there are more males per female at the beginning of the breeding season, was explained by the fact that newt males visit the breeding wetlands earlier than females and therefore the sex ratio is slightly shifted in favor of males [35,36]. We presume that the sex ratio could be affected by environmental disturbances as well. The planned hydrological interventions in the Szaporca Old-Drava oxbow provided the opportunity to test the applicability of this method under changing conditions, both during water shortage and following water replenishment, to establish a basis for long-term monitoring. Our case study seeks to highlight the often-underestimated impact of fluctuating water levels on passive trapping outcomes, with potential relevance to other wetland habitats.

The aim of our work was to answer the following questions by using bottle traps: (1) what is the relative frequency of occurrence of individuals of newts and other aquatic vertebrate species? (2) does the duration of trapping affect their efficiency and capture success? (3) does the efficiency and capture success of bottle traps change with increasing water volume? (4) do these changes affect the sex ratio of newts?

## 2. Materials and Methods

### 2.1. Study Area and Sampling Sites

**Figure 1:**
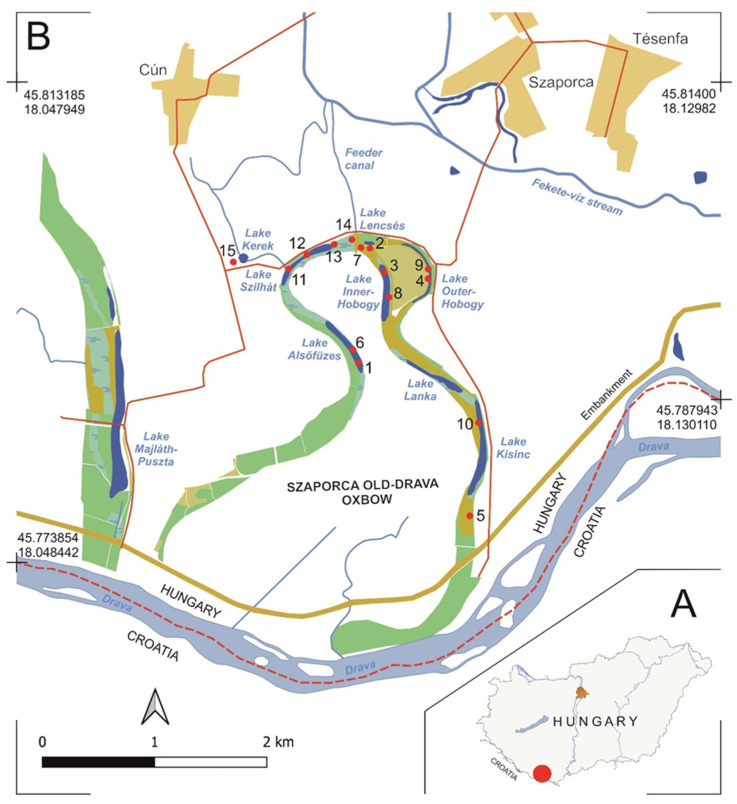
Location of the study area (red dot) in southern Hungary (**A**), and the current state of the Szaporca Old-Drava oxbow with the remaining lakes in its deeper sections, and with the 15 sampling sites (numbered red dots) where the bottle trap surveys were conducted (**B**). Map by Z. Ujvári.

The Szaporca Old-Drava oxbow is in the southern part of Hungary, in Baranya County, near the Hungarian Croatian state border, on the left bank of the Drava River, in the area of the municipalities of Cún and Szaporca (Figure 1).

Over the centuries, due to the process of succession, smaller lakes have formed in the deeper parts of its bed, and therefore it is often called the Cún-Szaporca oxbow system. Currently, six lakes can be distinguished (Figure 1) and there is also a nearby small lake with circular shape, which is connected to the oxbow with a canal. The lakes are in different phases of hydro-seral succession, which is accelerated by lowering groundwater levels, and can be traced in the changes of vegetation [39,40,41,42,43].

Various types of habitats are recorded in the study area, here we only mention the main vegetation types with some abundant plant species recorded near the sampling sites: euhydrophyte: floating, e.g., common duckweed (*Lemna minor*) and greater duckweed (*Spirodela polyrrhiza*); rooted aquatic vegetation, e.g., water aloe (*Stratiotes aloides*), water lily (*Nymphaea alba*) and water chestnut (*Trapa natans*); reed beds, e.g., reed (*Phragmites australis*), bulrush (*Typha angustifolia*, *T. latifolia*) and common club-rush (*Schoenoplectus lacustris*); reed meadowgrass (*Glyceria maxima*) and branched burreed (*Sparganium erectum*); tall sedge beds, e.g., cyperus sedge (*Carex pseudocyperus*) and tussock sedge (*Carex elata*); willow thickets (Calamagrostio-Salicetum cinereae); riparian forests: willow-poplar and alder groves, e.g., Leucojo aestivi-Salicetum albae; riparian weed and marsh vegetation on muddy banks.

A brief description of the lakes is provided based on the literature of the 1990s [34,35] and the current conditions experienced at the sampling sites:

Lake Alsófüzes, also known as Sárga-víz, which Pálfai [39] mistakenly calls Lake Lanka is in the western part of the oxbow system. It is 1 km long, 40 m wide on average, with water depth of 0.5 m and an open water surface of approximately 4 hectares. During the year of water shortage, traps were set in the marshy zone; following water replenishment, they were placed on the muddy bottom of the flooded hardwood forest.

Lake Szilhát is in the northwest, its water surface is 0.8 km long, its average width is 80 m, its depth is 1 m, its area is 6 hectares, and its water volume is 30,000 m^3^. The lake shore is covered with dense willow thickets, making access to the water difficult.

Lake Lencsés is the local name for the small lake located between lakes Szilháti and Inner-Hobogy. It has an area of only about one hectare, surrounded by a forest belt, and its steep shore is overgrown with willow thickets.

Lake Inner-Hobogy, also known as Kishobogy, is 1.2 km long, 40–50 m wide lake, with a surface of 6 hectares, a water depth of 1 m, and a water volume of 30,000 m^3^. On the shallow shore, marsh vegetation develops and occasionally merges with reed beds, cattail stands, and tall sedges.

Lake Outer-Hobogy is located east of Inner-Hobogy. Together, these two water bodies and their marshes cover 20 hectares. Floating vegetation has decreased, while rooted aquatic plants, like water aloe have expanded over a large area and a significant part has become silted. Extensive willow thickets and hardwood forest shrub layer dominate the edges.

Lake Lanka is located south of Lake Outer-Hobogy. It lacks free water surface, often dries up, and its silted-up bed is covered with reedbeds and willow thickets. Currently, it cannot be separated from Lake Kisinc, which lies south of it.

Lake Kisinc is the largest lake in the oxbow: 2 km long, 100 m wide on average, covering an area of 20 hectares, with water depth of 1 m, and a water volume of 200,000 m^3^. The main species of aquatic vegetation are rough pondweed (*Ceratophyllum demersum*), water lily, and water aloe. There are extensive reedbeds and bulrush associations, while a small area is covered by sedge beds. The lake is surrounded by willow thickets and white willow riparian forest. Here, the lake water level fluctuation was recorded by automatic data logger (type: DAS LRB 122, Dataqua Kft, Balatonalmádi, Hungary), logging was set 30 min frequency.

Lake Kerek was likely formed by clay-pit excavation. Its area is about one hectare, the depth is 1.5 m, and the open water surface is covered with floating aquatic vegetation. The lake is surrounded by reed beds and sedges.

The natural values and significance of the Szaporca Old Drava oxbow were recognized early. In 1962 steps were initiated to declare it a protected area [44] and it was officially designated a nature reserve in 1969 [40]. In 1979 it was included in the Ramsar List of Wetlands of International Importance (Ramsar code: 3HU001) [45]. It belongs to the Eastern Drava Natura 2000 Site (code: HUDD20007) [46] and it is part of the Danube-Drava National Park established in 1996 [44]. Since 2021 it has been part of the UNESCO designated area Mura-Drava-Danube Transboundary Biosphere Reserve [47].

**Figure 2:**
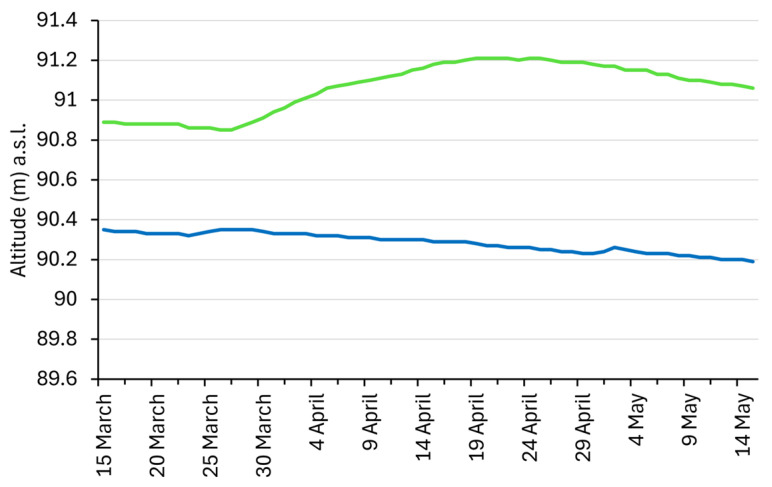
Changes in the water level of the Szaporca Old-Drava oxbow between 15 March and 15 May 2020 (blue line) and 2021 (green line) based on automatic measurements taken at the Cún 4 well.

Water supply to the Szaporca Old-Drava oxbow from the Drava could not be ensured due to the riverbed incision and the decrease in groundwater levels; therefore, an alternative solution had to be found. North of the oxbow, a tributary of the Drava, the Fekete-víz stream, is dammed to a level sufficient to supply a feeder canal which delivers 0.4 m^3^/s of water to the oxbow during two to three seasons per year [48]. On average 30,000 m^3^ of water can be delivered daily to the oxbow via the canal. However, it was found that the planned 20-day replenishment period is not sufficient to reach the optimal water level of Lake Kisinc (91.5 m a.s.l.) [48]. The dredging of the Lake Kisinc was planned during autumn, with the aim of starting the filling of the oxbow bed in the spring of 2021. This provided an excellent opportunity to carry out our planned study before and after the hydrological restoration. Evaporation from the water surface, transpiration from the lake shore vegetation and seepage into the groundwater significantly limit the effectiveness of the intervention [21]. To monitor the effectiveness of the water replenishment, a system was developed to model the relationship between the oxbow bed’s water capacity (m^3^), water level (m a.s.l.), and surface area covered by water (m^2^), based on groundwater levels measured in wells installed adjacent to the oxbow [48]. In early spring of 2000, water level was low in the oxbow, the estimated water volume was around 200,000 m^3^. Due to the water replenishment, the water level in the automatically measured well next to the oxbow and thus in the oxbow bed increased by almost one meter in 2021 compared to the previous year (Figure 2).

**Figure 3:**
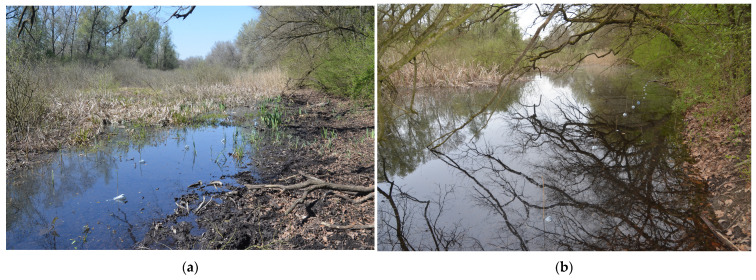
The shoreline zone of Lake Alsófüzes where the bottle traps were placed: (**a**) during the water-deficient period on 6 April 2020; (**b**) after water replenishment on 11 April 2021. Photos by J.J. Purger.

The estimated water volume on 7 April 2020 was 220,000 m^3^ and covered an area of about 210,000 m^2^. In contrast, by water replenishment achieved exactly one year later, on 7 April 2021, the estimated water volume had reached 270,000 m^3^ and covered an area of about 420,000 m^2^. These changes were most visible along the shore of the oxbow where the bottle traps were placed (Figure 3).

### 2.2. Bottle Trap Survey

We used funnel traps made of plastic bottles to capture newts, as this is the recommended method within the framework of the Biodiversity Monitoring System in Hungary [33]. Due to the simplicity and low costs of the method [32], it has become one of the most widely used, important tools for species surveys and monitoring [49,50,51,52,53,54,55]. Although other methods can be more efficient in monitoring of newts [52,53], bottle traps offer the additional advantage of requiring minimal space, allowing deployment even in dense reed beds. They are suitable not only for detecting newts but also other aquatic vertebrates, like frogs, fish and snakes [34]. The method is labor-intensive, but less dependent on weather conditions, causes no mortality, and can be standardized. Relative population changes can be easily tracked using capture results [56,57]. The capture success of bottle traps is usually higher than their capture efficiency, as some traps can catch more than one newt. Therefore, these values do not overlap and do not indicate sampling efficiency but can be used to compare results obtained with standardized sampling (e.g., 100 traps/day). In our studies we used bottle traps made of colorless, transparent 1.5-L plastic bottles. At each sampling site, 25 bottle traps were placed along a 25-m transect, one meter apart in 25–30 cm deep water, with their openings always facing the bottom, towards the center of the lake. We paid special attention to ensuring that the air trapped inside the bottle remained in contact with the external environment, so that the traps could operate throughout the day. Bottle trapping lasted for four days in 2020 and 2021, so the number of animals captured at each site refers to 100 trap days [57,58].

In 2020, the survey was conducted at five sampling sites (1–5) from 3 to 7 April, thus realizing 500 trap days. In 2021, we repeated the survey at the existing five sampling sites (1–5) from 7 to 11 April, as well as operating the same number of traps at five additional sites (6–10) in the same lakes. In this way, we doubled the number of trap days (1000) in 2021 (when the water was replenished) for comparison to 2020 (when there was a water shortage in the oxbow). Additionally, in 2021 we placed traps at four sampling sites in Lake Szilhát and one in the nearby Lake Kerek (sampling sites 11–15), thus realizing an additional 500 trap days (Supplementary Materials Table S1). Assuming that the number of newts living in the lakes is constant, if the amount of water in the oxbow is significantly increased, twice or even three times as many traps would be needed to detect them in the same ratio.

Traps were checked every day between 10:00 and 14:00. Animals were handled using silicone gloves, which were changed at each sampling site to avoid the possibility of spreading any possible infections. We photographed the uniquely patterned ventral side of each captured newt individual, either while holding it in our hands, or placed in an aquarium or in a Petri dish [50], so that if they were recaptured, we did not take them into account. We also identified the sex of the newts to determine the sex ratio based on the number of males and females (Supplementary Materials Figure S1). After completing the study, we collected and counted all the tools we used, then cleaned and dried them and placed them in the storage room of the national park visitor center.

### 2.3. Monitoring

Based on the results of our baseline study conducted in 2020–2021, we performed additional data collection in 2024 and 2025 aiming to test a simple and low-cost monitoring plan for the National Park. We operated 25 bottle traps for two days at the previously designated sampling sites 1 and 6 in the Lake Alsófüzes and 4 and 9 in the Lake Outer- Hobogy, thus standardizing the monitoring efforts to 100 trap days per lake (Figure 1, Supplementary Materials Table S1). Surveys were conducted from 8–10 April in 2024 and 6–8 April in 2025.

### 2.4. Data Analysis

Bottle trap efficiency is defined as the number (*n*) or percentage (%) of the bottle traps that captured smooth newt individuals. Capture success indicates the number of newts caught by all bottle traps (*n*) or the relative frequency (%) of newts. We used chi-square (χ^2^) goodness of fit for more than two categories to test whether the efficiency and capture success of the bottle traps change significantly in the days following their placement [59]. For this test, we used results from 2000 trap days: 500 bottle traps operated all four days. In this case, we considered the results of both years because we assumed that the number of newts in the oxbow did not change significantly over two consecutive years.

To determine whether the efficiency and capture success of the traps differed during periods of water scarcity and replenishment, we used test of chi-square goodness of fit for two categories [59]. In this case, the values used in the comparison always refer to five samples, or 500 trap days. In this case, we compared the results of 500 trap days in 2020 with the results of 500 trap days in 2021 at the same sampling locations, then with the results of 500 trap days in the same lakes but at different locations, and finally with the results of 500 trap days in other lakes connected to the oxbow.

In addition to determining the ratio of male and female smooth newts, we also tested whether the number of individuals of the two sexes changed significantly during the two years of study based on the capture results. In the first year, 500 trap days were realized; therefore, the results of the 1500 trap days realized in the second year were also standardized to 500 trap days. The standardization was necessary to compare the results of the same trapping days in the two years. To compare the results, chi-square goodness of fit for two categories was used. The two-tailed *p* significance level was *p* < 0.05 in all cases [59].

## 3. Results

### 3.1. Vertebrate Species Detected Using Bottle Traps

Individuals of three fish species, two newt species, and two frog species were detected using bottle traps in the Szaporca Old Dráva oxbow in the spring of 2020 and 2021 (Table 1, Supplementary Materials Tables S2 and S3). Among fish, the European weather loach *Misgurnus fossilis* (Linnaeus, 1758) was found in four lakes (Alsófüzes, Lencsés, Inner-Hobogy, Kisinc) and the European mudminnow *Umbra krameri* Walbaum, 1792 was also detected in four lakes (Alsófüzes, Lencsés, Inner-Hobogy, Outer-Hobogy), while two individuals of the Danube whitefin gudgeon *Romanogobio vladykovi* (Fang, 1943) were caught only in Lake Szilhát. Based on the number of captures, the smooth newt, which occurs in all lakes of the oxbow system, was the most common (Table 1). However, we only caught two individuals of the Danube crested newt (a male in 2020 in Lake Alsófüzes and a female in 2021 in Lake Szilhát). One individual of common spadefoot toad *Pelobates fuscus* (Laurenti, 1768) was found in Lake Outer-Hobogy, and edible frogs *Pelophylax* kl. *esculentus* (Linnaeus, 1758) were caught in bottle traps in all lakes except Lake Kisinc and Lake Kerek.

Based on the results, with 100 bottle traps, only the smooth newt can be detected with a relative frequency of 10%, while the chance of other vertebrate species being trapped is <1% (Table 1). Therefore, when answering the questions formulated in the objectives, we only used data related to the smooth newt.

### 3.2. The Efficiency and Capture Success of Bottle Traps

**Figure 4:**
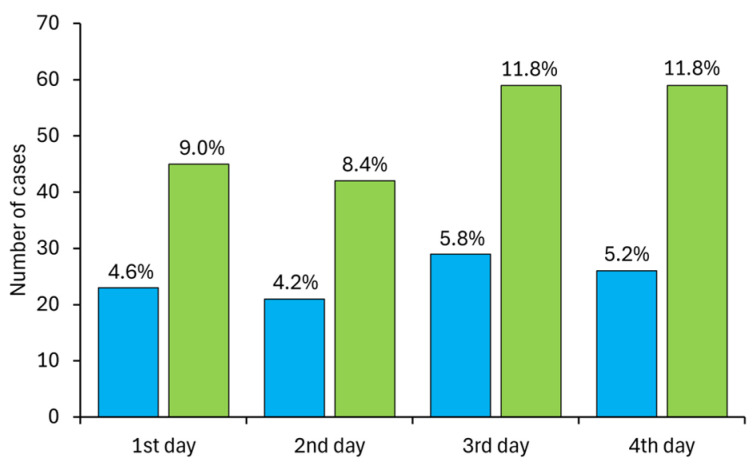
The efficiency of bottle traps (blue bars) and their capture success of smooth newts (green bars) in four days after their placement.

The bottle traps operated with similar efficiency on the days following their placement (4–6%), as the number of traps that caught smooth newts (23, 21, 29, 26) did not show significant differences (χ^2^ = 1.48, df = 3, *p* = 0.686) over the days (Figure 4).

Bottle trap efficiency was lower than a capture success since a single trap often captured several smooth newts (Table 2). The capture success of the bottle traps on the days following their placement (8–12%), as the number of smooth newts captured (45, 42, 59, 59) did not show significant difference (χ^2^ = 4.78, df = 3, *p* = 0.189) over the days (Figure 4).

### 3.3. The Effect of Water Conditions on the Efficiency and Capture Success of Bottle Traps

In the spring of 2020, when the water was scarce in the oxbow, the efficiency of bottle traps was 10.4% (Table 2). The following year (2021), when water volume increased significantly and covered twice the area as before, the efficiency of bottle traps was only 3% at the same sampling sites (1–5), and 5.6% at the other five sampling sites (6–10) (Table 2). At the other five sampling sites (11–15) designated in Lake Szilhát and Lake Kerek, the efficiency of bottle traps was only 0.8% (Table 2). During the control, 75% of the bottle traps that caught smooth newts contained only one or two individuals, but in several cases, more (3–7) individuals were found in one trap at the same time (Table 2).

The efficiency of bottle traps decreased significantly after increasing the water volume (2021) regardless of the lake in which they were placed (Table 3). The results obtained in 2020 (collected at sampling sites 1–5) in the first row of the table were compared with the results obtained in 2021, but the latter were arranged into three data sets (sampling sites 1–5, 6–10, 11–15, respectively). This was necessary so that we could compare the 2020 survey with the results obtained in 2021 with different sample sizes.

However, when comparing the number of smooth newts caught by the traps (capture success), a somewhat different pattern emerged. At the 5 locations selected in 2020 (1–5) during low water levels, the number of smooth newts captured with bottle traps (*n* = 85) was significantly higher than at the same locations (1–5) in 2021 (*n* = 37), and this trend was also observed at five additional sampling sites (11–15) in two other lakes in 2021, where only six individuals were captured. However, when the number of newts captured in 2020 (*n* = 85) was compared with the number of those captured in the same lakes but at five other sampling sites (06–10) (*n* = 77), the difference was no longer statistically significant (Table 3).

Comparing the efficiency of traps operating at the five sampling sites (1–5) in the spring of 2020 with the efficiency of the doubled sample (1–10) and trap number in 2021, the efficiency of the traps remained similar (χ*^2^* = 0.85, df = 1, *p* = 0.356). In contrast, the capture success—measured by the number of smooth newts caught—increased significantly from 85 individuals in 2020 to 114 in 2021 (χ*^2^* = 4.23, df = 1, *p* < 0.05). The number of smooth newts captured with traps changed between the two years. In 2021, traps placed in Lake Alsófüzes alone captured more individuals (*n* = 89) than all traps across all lakes in 2020 combined (*n* = 85).

### 3.4. Sex Ratio of Smooth Newts

In both years, most smooth newt individuals captured by bottle traps in the oxbow lakes were males (Table 4). In 2020, 73% of smooth newts caught with traps were male and 27% were female, while in 2021, 87.5% were male and 12.5% were female. In the first year, there was 1 female to 2.7 males, in the second year this ratio shifted even more in favor of males to 1:7. Using the standardized sample size (500 trap days), the number of males caught in traps in the water-scarce year (2020) was significantly higher (χ^2^ = 7.51, df = 1, *p* < 0.01) than in the following water-replanished year (2021). Significantly more females were caught in traps in 2020 than in 2021 (χ^2^ = 11.57, df = 1, *p* < 0.001).

### 3.5. First Results of Monitoring

In 2024 we used 100 bottle traps and detected two individuals of the Danube crested newt in Lake Alsófüzes. More smooth newts were captured in 2025 when 11 individuals were recorded in Lake Alsófüzes, with a sex ratio of 2.7:1 in favor of males. The bottle traps also caught individuals of the crucian carp *Carassius carassius* (Linnaeus, 1758), and the pumpkinseed *Lepomis gibbosus* (Linnaeus, 1758), as well as the fire-bellied toad *Bombina bombina* (Linnaeus, 1761), which were not detected with this method during the baseline surveys in 2020 and 2021 (Supplementary Materials Table S4).

## 4. Discussion

In this study we used the bottle traps primarily to capture newts, and we found both species known to occur in the study area: smooth newt, the most common caudatan amphibian in Hungary [25,30], and the Danube crested newt, which is much rarer, with limited occurrence data in the South Transdanubia region [30,31]. We also captured some rare aquatic vertebrates. Among the three fish species detected with bottle traps, the European weather loach was found in four lakes. These are considered new localities, as it had previously only been recorded in Lake Kisinc in 1996 and in Lake Szilhát in 2001 [28]. We found the individuals of the European mudminnow in four lakes, also representing new occurrences, as they were only known from Lake Szilhát in 2001 [28] and few sites on the Hungarian section of the Drava River [29]. Given recent records of the species in multiple Croatian sites [60], we presume that it inhabits other wetlands in the region. As a Ramsar site, the Szaporca Old-Drava oxbow can play an important role in the conservation of these two protected fish species through continued water replenishment. We also recorded a Danube whitefin gudgeon, a protected fish species which was not previously reported from the oxbow. Two individuals of this flow-preferring species were captured by bottle traps in the part of Lake Szilhát where the feeder canal with strong water flow provides the water replenishment in the oxbow from Fekete-víz stream. The occurrence of Danube whitefin gudgeon in the Fekete-víz stream was already detected in 1999 [28]. The feeder canal can also be a possible route for the spread of invasive species, highlighting the importance of ongoing monitoring. We also confirmed the occurrence of the common spadefoot toad, which has been known from the Szaporca area and along the Drava [61]. Several individuals of the edible frog were found at all sampling sites since it is a common dominant frog species in wetlands [30]. Except for the smooth newt, the other vertebrate species were captured in small numbers, suggesting that their detection was made possible only due to the high number of traps used.

The efficiency of bottle traps remained consistently low over consecutive days, and at the same time, the number of smooth newts captured did not change significantly. This result is important because if the abundance of newts in a wetland is higher, it will also be reflected in the efficiency and capture success of bottle traps. This implies that both the number of traps and trapping duration can be adapted to habitat conditions. For example, 25 traps deployed for four days, or 50 traps deployed for two days both result in 100 trap days, offering flexibility while maintaining standardization for comparative purposes. If the size of the habitat or its accessibility do not allow the use of a larger number of bottle traps, then the survey can be prolonged for several days. In our study, 25 traps were operated at each sampling site for four days, but this can also be solved by placing 50 traps at one site and operating them for two days, thus realizing 100 trap days in both cases. Standardizing the surveys makes it possible to compare the results. The efficiency and capture success of bottle traps can vary substantially depending on the density of newts within the oxbow. If the newt population does not change significantly between two trapping events (e.g., two consecutive years), but the water volume in the oxbow increases significantly, the efficiency and capture success of the bottle traps will decrease. In such cases, it is advisable to increase the number of traps proportionally to the increase in water volume to achieve more realistic results. At low water levels, the number of smooth newts caught with bottle traps was similar in the four lakes (Alsófüzes, Lencsés, Inner-Hobogy and Outer-Hobogy). In the following year when water replenishment was started, trapping results changed markedly: in Lake Alsófüzes the traps captured significantly more smooth newts than in the other three sites. One possible explanation for this is that Lake Alsófüzes is less affected by water flow than other lakes of the oxbow (water arriving through the feeder canal, flowing through the eastern edge of Szilhát, Lencsés then Inner- and partly Outer-Hobogy, reaches Lake Kisinc via Lake Lanka). The water flow in Alsófüzes is thus barely perceptible, and the water level only starts to rise when the lower lakes of the oxbow system are already full. This assumption is difficult to prove, but we know that smooth newts prefer stagnant waters [30] and can travel longer distances [62]. Their possible migration to the less disturbed Lake Alsófüzes cannot be excluded. It is important to note that in the spring of 2020, due to low water level, sampling was conducted in separate lakes of the oxbow, while in 2021, due to water replenishment, sampling took place in a single, unified water body. Only one smooth newt was captured in Lake Kisinc, which could have been caused not only by the disturbance caused by water replenishment, but also by the previous mud dredging and the introduction of predatory fish. The last lake in the oxbow system with the deepest and largest free water surface is also used by anglers [40,41]. After our first year of study was conducted, we were informed that the local fishing association had not only stocked the usual annual 1.0–1.5 tons of carp in Lake Kisinc but also released about 2000 pike fry into the lake not far from our sampling site. In the first and the subsequent years of our study, small pikes could be seen almost everywhere in the shallow water among the vegetation (G. Wágner, personal observation). Several studies have shown that the development of newt populations is also influenced by the population of fish that feed on them and their eggs and larvae [63,64,65]. Our results highlight both the direct and indirect impacts of water replenishment, not only on smooth newts but also on other vertebrate populations.

In the Szaporca Old Drava oxbow, the male-to-female ratio of smooth newt considerably increased by water replenishment. Our survey was performed in early April, when individuals of both sexes should have already been in the water for a long time, so the shift in the sex ratio in favor of males cannot be explained by their earlier arrival at the breeding site [35,36,37,38]. In our study area the shift in the sex ratio could have been caused by other factors. Water replenishment raised the water level by nearly one meter resulting in partial inundation of terrestrial vegetation and altering the structure of shoreline habitats. In the case of less steep shore sections, aquatic plants and reed grew near the shore in shallow water, and as water rose, traps were placed on bare or litter-covered substrates. In those steeper shore sections, traps were not placed among the aquatic vegetation but on the terrestrial vegetation due to the raised water level. The breeding grounds of smooth newts are usually wetlands overgrown with rich vegetation [66]. Additionally, macrophytes can reduce the capture success of newt [55]. In the second year of our study, 74% of the smooth newts were caught in the traps in Lake Alsófüzes. Here, due to the rise in water level, there were no plants in the coastal zone where the traps were located, only litter. It is conceivable that male smooth newts, since they are not territorial, were more likely to be caught in the traps [67] than females, who prefer to stay among aquatic plants [66]. We assume that habitat changes caused by water replenishment may have also played a role in the traps catching more males than females.

It is noteworthy that in 2024 a short 100 trap-day survey in Lake Alsófüzes yielded two Danube crested newts—the same number of this rare species caught during the entire 2000 trap-day baseline—although, unexpectedly, no smooth newts were caught in that survey. These preliminary results currently lack explanation and require further study. The bottle traps caught European mudminnow in Lake Alsófüzes in both years (2024, 2025), which is a remarkable result and highlights the need of monitoring. The isolated populations of this endemic fish species of the Middle Danube show a metapopulation structure, so reliable data are needed for their long-term maintenance [68]. This is particularly relevant currently, since another predatory food competitor, the Amur sleeper *Perccottus glenii* Dybowski, 1877, is an invasive predatory species known for rapid population outbreaks and severe ecological impact, thus threatening the European mudminnow populations not only by reducing the food source, but also by its predation activity [69,70]. The spread of non-native species like the Amur sleeper accelerates freshwater ecosystem degradation and increases extinction risks for native fauna [18]. To our knowledge, the Amur sleeper has not yet appeared in the oxbow, which is why it is important to confirm the current presence of the European mudminnow. The fact that with a minimal number of bottle traps, individuals of vertebrate species were detected in a few days, which were not detected during twenty times as many trap days during the baseline study, suggests that water replenishment has substantially reshaped shoreline habitats. Topography, substrate structure, and current vegetation conditions likely influenced these outcomes. In future monitoring efforts, we recommend also placing bottle traps in the feeder canal to enable early detection of invasive species before they establish in the oxbow system.

The Szaporca Old-Drava oxbow will hopefully be able to maintain its valuable wildlife for a long time, further increasing the diversity of the riverside habitats, due to the favorable habitat conditions created by water replenishment [71]. The results of our study pointed out that monitoring of the wildlife is necessary, ideally continued within the framework of the Hungarian Biodiversity Monitoring System [57].

## 5. Conclusions

Using bottle traps can provide important data on the occurrence of rare aquatic vertebrates in wetlands. However, their suitability for long-term monitoring is limited due to variability in efficiency and capture success, which can be influenced by environmental factors and unknown conditions.

Our results indicate that bottle trap efficiency and capture success did not change significantly over consecutive days, suggesting that both the number of traps and their deployment duration can be optimized based on field conditions.

Importantly, fluctuations in water quantity significantly influenced both trap efficiency and capture success, potentially in more ways, for example, through vegetation shifts or predator presence, highlighting the need to consider hydrological conditions in future long-term monitoring programs.

## Figures and Tables

**Table 1:** Number of individuals (*n*) and relative frequency (%) of fish, newt and frog species captured with bottle traps (during 2000 trap days) in the lakes of the Szaporca Old Drava oxbow in the spring of 2020 and 2021.

Vertebrate Species	*n*	%
European weather loach *Misgurnus fossilis* (Linnaeus, 1758)	7	0.35
European mudminnow *Umbra krameri* Walbaum, 1792	11	0.55
Danube whitefin gudgeon *Romanogobio vladykovi* (Fang, 1943)	2	0.1
Smooth newt *Lissotriton vulgaris* (Linnaeus, 1758)	205	10.25
Danube crested newt *Triturus dobrogicus* (Kiritzescu, 1903)	2	0.1
Common spadefoot toad *Pelobates fuscus* (Laurenti, 1768)	1	0.05
Edible frog *Pelophylax* kl. *esculentus* (Linnaeus, 1758)	10	0.5
Total (Σ)	238	11.9

**Table 2:** The number of bottle traps according to how many (1–7) smooth newts they captured at each time during the control.

Year _(Sites)_	Trap	1	2	3	4	5	6	7	Σ	%
2020 _(1–5)_	500	33	11	5	1	1	1	0	52	10.4
2021 _(1–5)_	500	4	7	1	1	1	0	1	15	3.0
2021 _(6–10)_	500	4	11	7	1	4	1	0	28	5.6
2021 _(11–15)_	500	2	2	0	0	0	0	0	4	0.8
Σ Bottle Trap	2000	43	31	13	3	6	2	1	99	4.9
Σ Smooth Newt		43	62	39	12	30	12	7	205	10.2

**Table 3:** Comparison of the number of bottle traps that caught smooth newts (bottle trap efficiency, left part of table) and the number of smooth newts (capture success, right part of table): in the water-scarce year 2020 vs. the water-rich year 2021 (500 traps at 5 sampling sites each time).

	Bottle Trap: 2020 _(1–5)_ *n* = 52				Smooth Newt: 2020 _(1–5)_ *n* = 85			
Year _(Sites)_	*n*	χ^2^	df	*p*	*n*	χ^2^	df	*p*
2021 _(1–5)_	15	20.43	1	<0.001	37	18.88	1	<0.001
2021 _(6–10)_	28	7.20	1	<0.01	77	0.39	1	0.529
2021 _(11–15)_	4	41.14	1	<0.001	6	68.58	1	<0.001

**Table 4:** Sex ratio of smooth newts in the lakes of the Szaporca Old-Drava oxbow in 2020 and 2021.

Year	2020		2021	
Sex	Male	Female	Male	Female
Lake Alsófüzes	14	3	81	8
Lake Lencsés	16	4	9	1
Lake Inner-Hobogy	20	7	5	4
Lake Outer-Hobogy	12	8	6	-
Lake Kisinc	-	1	-	-
Lake Szilhát	-	-	4	1
Lake Kerek	-	-	-	1
Total (Σ)	62	23	105	15

## Data Availability

The original contributions presented in this study are included in the article/Supplementary Material. Further inquiries can be directed to the corresponding author(s).

## Supplementary Materials

The following supporting information can be downloaded at: https://www.mdpi.com/article/10.3390/biology14101416/s1, Table S1: Sampling sites in the Szaporca Old-Drava oxbow with the coordinates (°), altitudes (m) and the number of trap days realized in the years of the study; Table S2: Results of studies conducted with bottle traps in the Szaporca Old-Drava oxbow in 2020 and 2021 on five sampling sites: 1-Lake Alsófüzes; 2-Lake Lencsés; 3-Lake Inner-Hobogy; 4-Lake Outer-Hobogy; 5-Lake Kisinc (25 bottle traps operated at each sampling site for 4 days, the capture results refer to 100 trap days); Table S3: Results of studies conducted with bottle traps in the Szaporca Old-Drava oxbow oxbow in 2021 on ten sampling sites: 6-Lake Alsófüzes; 7-Lake Lencsés; 8-Lake Inner-Hobogy; 9-Lake Outer-Hobogy; 10-Lake Kisinc; 11–14-Lake Szilhát; 15-Lake Kerek (25 bottle traps operated at each sampling site for 4 days, the capture results refer to 100 trap days); Table S4: Results of monitoring on two lakes of Szaporca Old-Drava oxbow conducted in 8–10 April 2024 and 6–8 April 2025 (m-male, f-female); Figure S1: We photographed the uniquely patterned ventral side of each captured newt. Top image: male and female smooth newt photographed in an aquarium (Photo by C. Fekete) Middle: male Danube crested newt photographed in a Petri dish (Photo by J.J. Purger). Bottom image: female Danube crested newt photographed in hand (Photo by J.J. Purger).
