# Supplementary material for: Methodological Pitfalls of Monitoring: Water Conditions Affect the Efficiency of Bottle Traps and Capture Success
Source: Biology (Basel). 2025 Oct 15;14(10):1416. doi: 10.3390/biology14101416 (PMC12561506; doi:10.3390/biology14101416)
Supplement: Supplementary file 1 [file biology-14-01416-s001.zip › biology-3841157-supplementary.pdf]

Table S1. Sampling sites in the Szaporca Old-Drava oxbow with the coordinates (°), altitudes (m) and the number of trap days realized in the years of the study.

Locality	No	Latitude	Longtude	Altitude	2020	2021	2024	2025
Lake Alsófüzes	1.	45.790710	18.088211	91	100	100	50	50
(Alsófüzesi-tó)	6.	45.791816	18.087487	93		100	50	50
Lake Lencsés	2.	45.800273	18.089076	96	100	100		
(Lencsés-tó)	7.	45.800283	18.088482	93		100		
Lake Inner-Hobogy	3.	45.798132	18.091033	92	100	100		
(Belső-Hobogy)	8.	45.796189	18.091592	92		100		
Lake Outer-Hobogy	4.	45.797817	18.096156	93	100	100	50	50
(Külső-Hobogy)	9.	45.798359	18.096055	94		100	50	50
Lake Kisinc	5.	45.778369	18.101395	90	100	100		
(Kisinci-tó)	10.	45.785928	18.102440	89		100		
Lake Szilhát	11.	45.798277	18.079829	92		100		
(Szilháti-tó)	12.	45.799574	18.081995	92		100		
	13.	45.800397	18.085185	92		100		
	14.	45.800667	18.087318	92		100		
Lake Kerek (Kerek-tó)	15.	45.798443	18.072863	90		100		
Total (Σ)					500	1500	200	200

Table S2. Results of studies conducted with bottle traps in the Szaporca Old-Drava oxbow in 2020 and 2021 on five sampling sites: 1- Lake Alsófüzes; 2- Lake Lencsés; 3-Lake Inner-Hobogy; 4- Lake Outer-Hobogy; 5-Lake Kisinc (25 bottle traps operated at each sampling site for 4 days, the capture results refer to 100 trap days)

**Date**	**3-7 April 2020**					**7-11 April 2021**				
**Samping site**	**1.**	**2.**	**3.**	**4.**	**5.**	**1.**	**2.**	**3.**	**4.**	**5.**
Misgurnus fossilis	1				2		2			
Umbra krameri	2	1	2	1		1	2			
Romanogobio vladykovi										
Lissotriton vulgaris	17	20	27	20	1	33		1	3	
Triturus dobrogicus	1									
Pelobates fuscus									1	
Rana kl. *esculentus*	1		2	1		1		1		
Total (Σ)	22	21	31	22	3	35	4	2	4	0

Table S3. Results of studies conducted with bottle traps in the Szaporca Old-Drava oxbow oxbow in 2021 on ten sampling sites: 6- Lake Alsófüzes; 7- Lake Lencsés; 8-Lake Inner-Hobogy; 9- Lake Outer-Hobogy; 10-Lake Kisinc; 11-14-Lake Szilhát; 15-Lake Kerek (25 bottle traps operated at each sampling site for 4 days, the capture results refer to 100 trap days)

**Date**	**7-11 April 2021**									
**Sampling site**	**6.**	**7.**	**8.**	**9.**	**10.**	**11.**	**12.**	**13.**	**14.**	**15.**
Misgurnus fossilis			2							
Umbra krameri	1		1							
Romanogobio vladykovi									2	
Lissotriton vulgaris	56	10	8	3		2	3			1
Triturus dobrogicus						1				
Pelobates fuscus										
Rana kl. *esculentus*		1		2				1		
Total (Σ)	57	11	11	5	0	3	3	1	2	1

Table S4. Results of monitoring on two lakes of Szaporca Old-Drava oxbow conducted in 8-10 April 2024 and 6-8 April 2025 (m-male, f-female)

**Locality**	**Lake Alsófüzesi**				**Lake Outer-Hobogy**			
**Year**	**2024**	**2024**	**2025**	**2025**	**2024**	**2024**	**2025**	**2025**
**Sampling site**	**1.**	**6.**	**1.**	**6.**	**4.**	**9.**	**4.**	**9.**
Carassius carassius					1	1		
Umbra krameri		1	1	1				
Lepomis gibbosus	1							
Lissotriton vulgaris			8m+2f	1f				
Triturus dobrogicus		1m+1f						
Bombina bombina					1			
Total (Σ)	1	3	11	2	2	1	0	0

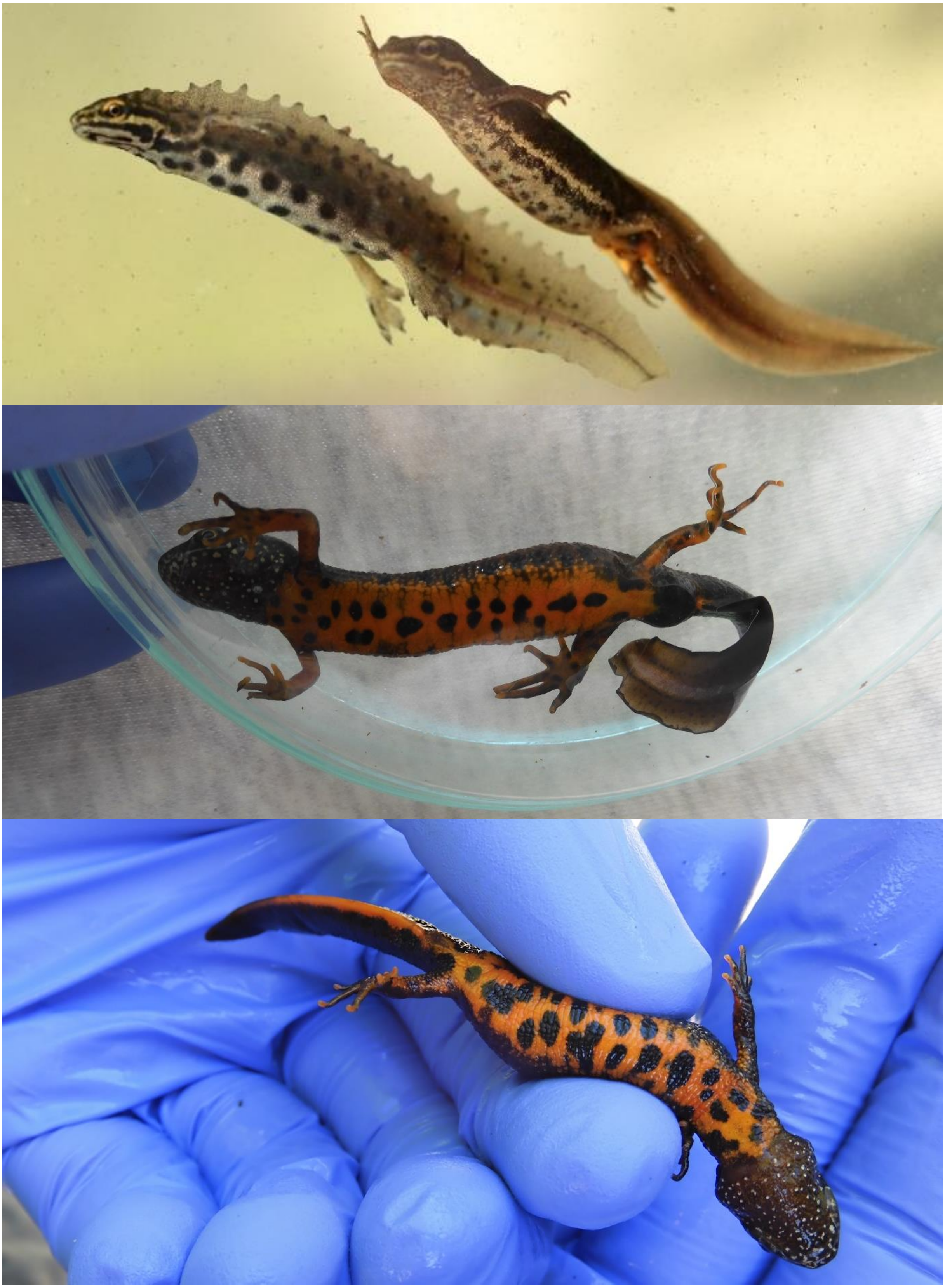

Figure S1. We photographed the uniquely patterned ventral side of each captured newt. Top image: male and female smooth newt photographed in an aquarium (Photo by C. Fekete) Middle: male Danube crested newt photographed in a Petri dish (Photo by J.J. Purger). Bottom image: female Danube crested newt photographed in hand (Photo by J.J. Purger).
